# Different mechanisms contributing to savings and anterograde interference are impaired in Parkinson's disease

**DOI:** 10.3389/fnhum.2013.00055

**Published:** 2013-02-27

**Authors:** Li-Ann Leow, Aymar de Rugy, Andrea M. Loftus, Geoff Hammond

**Affiliations:** School of Psychology, University of Western AustraliaCrawley, WA, Australia

**Keywords:** visuomotor rotation, motor learning, motor adaptation, anterograde interference, savings, Parkinson's disease

## Abstract

Reinforcement and use-dependent plasticity mechanisms have been proposed to be involved in both savings and anterograde interference in adaptation to a visuomotor rotation (cf. Huang et al., 2011). In Parkinson's disease (PD), dopamine dysfunction is known to impair reinforcement mechanisms, and could also affect use-dependent plasticity. Here, we assessed savings and anterograde interference in PD with an A1-B-A2 paradigm in which movement repetition was (1) favored by the use of a single-target, and (2) manipulated through the amount of initial training. PD patients and controls completed either limited or extended training in A1 where they adapted movement to a 30° counter-clockwise rotation of visual feedback of the movement trajectory, and then adapted to a 30° clockwise rotation in B. After subsequent washout, participants readapted to the first 30° counter-clockwise rotation in A2. Controls showed significant anterograde interference from A1 to B only after extended training, and significant A1-B-A2 savings after both limited and extended training. However, despite similar A1 adaptation to controls, PD patients showed neither anterograde interference nor savings. That extended training was necessary in controls to elicit anterograde interference but not savings suggests that savings and anterograde interference do not result from equal contributions of the same underlying mechanism(s). It is suggested that use-dependent plasticity mechanisms contributes to anterograde interference but not to savings, while reinforcement mechanisms contribute to both. As both savings and anterograde interference were impaired in PD, dopamine dysfunction in PD might impair both reinforcement and use-dependent plasticity mechanisms during adaptation to a visuomotor rotation.

## Introduction

Motor adaptation is the process through which the motor system alters movements to maintain performance in response to perturbations or changes in the state of the effector and/or of the environment. These perturbations evoke discrepancies between the predicted motor outcome and the actual motor outcome, which are thought to drive the iterative updating of an internal model that predicts the consequences of motor commands (i.e., a forward model; Thoroughman and Shadmehr, 1999). However, this internal-model based account of motor adaptation cannot fully explain persistent effects of initial learning on subsequent performance after the motor output is returned to the original, unadapted state (Huang et al., 2011). Persistent effects of initial learning can be evident in savings, when initial learning enhances subsequent adaptation to a similar perturbation, and in anterograde interference, when initial learning impairs subsequent adaptation to an opposing perturbation. These effects could be explained by a two-process model which posits a fast-learning, fast-forgetting process that occurs by updating an internal model, as well as a slow-learning, slow-forgetting process that does not occur by updating an internal model (Huang et al., 2011). Two mechanisms have been suggested for this “model-free” slow process: reinforcement learning, where repeatedly pairing the adapted movement with a rewarding outcome (e.g., hitting the target) reinforces that movement such that there would be a subsequent bias toward reselecting that movement, and use-dependent plasticity, where repetition alone of a particular movement (i.e., independently of a reward associated with the adaptation) would bias subsequent movements toward the repeated movement (Huang et al., 2011).

Savings is thought to occur through reinforcement mechanisms (Huang et al., 2011). Consistent with this proposal, savings is impaired in Parkinson's disease (PD) (Marinelli et al., 2009; Bedard and Sanes, 2011; Leow et al., 2012), a neurological disorder characterized by dysfunctional dopamine neurotransmission and consequent reinforcement learning deficits (Frank et al., 2004; Shohamy et al., 2006; Rutledge et al., 2009). Despite unimpaired initial learning where the rate and extent of error reduction is indistinguishable from that of controls, substantial deficits in savings have been repeatedly observed on PD patients using various protocols (Marinelli et al., 2009; Bedard and Sanes, 2011; Leow et al., 2012). Deficient savings is evident within the same test session (Bedard and Sanes, 2011; Leow et al., 2012), between test sessions separated by a 24-h delay (Marinelli et al., 2009; Bedard and Sanes, 2011; Leow et al., 2012), and during single-target (Leow et al., 2012) and multiple-target adaptation (Marinelli et al., 2009; Bedard and Sanes, 2011). In healthy adults, A1-B-A2 savings (i.e., savings in A2 after adapting to a first perturbation in A1 followed by an opposing perturbation in B) is also evident after extended training in A1, but not after limited training in A1 (Krakauer et al., 2005). A reinforcement interpretation suggests that with limited training, reinforcing the adapted movement for the A1 perturbation and subsequently reinforcing the adapted movement for the opposing B perturbation forms two competing movement-reward associations, which inhibits reselection of the A1-adapted movement in A2, thus preventing savings (Krakauer, 2009; Huang et al., 2011). In contrast, extended training in A1 strengthens the association of the A1 adapted movement with reward, increasing the bias to reselect it in A2, thus evoking savings. Anterograde interference may similarly be interpreted in terms of reinforcement: reinforcing a first adapted movement might bias the selection of that particular movement in subsequent learning of an opposing perturbation and cause interference (Huang et al., 2011). If reinforcement mechanisms contribute to A1-B-A2 savings and anterograde interference, then reinforcement learning deficits in PD should impair both A1-B-A2 savings and anterograde interference.

The role of use-dependent plasticity in savings and anterograde interference is unclear. Although previous studies suggest that use-dependent plasticity is neither necessary nor sufficient for savings (Huang et al., 2011), it might contribute to anterograde interference, which is typically measured in B without washing out movement biases induced by movement repetition in A1 (Sing and Smith, 2010). Like reinforcement learning, use-dependent plasticity is dopamine sensitive: the formation of use-dependent movement biases is accelerated by the dopamine precursor Levodopa in healthy adults (Floel, 2005; Floel et al., 2008), and is slowed by dopamine antagonists in schizophrenia patients (Daskalakis et al., 2008). While there is still no direct evidence that use-dependent plasticity is impaired in PD, it is likely to be affected by dysfunctional dopamine neurotransmission, and might thus impair anterograde interference in PD.

The present study examined A1-B-A2 savings and anterograde interference in PD patients and older adult controls. In A1, participants first adapted to a 30° counter-clockwise rotation of the visual feedback of the movement trajectory, with either limited (25 trials) or extended (80 trials) training. Subsequently in B, all participants completed a block of adaptation trials with a 30° clockwise rotation. After subsequent washout with veridical feedback trials, all participants re-adapted to the first 30° counter-clockwise rotation in A2. As dopamine dysfunction in PD could affect both reinforcement and use-dependent mechanisms, it was hypothesized that PD patients would show both impaired savings and impaired anterograde interference.

## Methods

### Participants

A total of 16 mild-to-moderate PD patients and 18 neurologically intact older adult controls who were naive to the experimental design were recruited from the Parkinson's Western Australia newsletter and local newspapers. This study was approved by the Human Research Ethics Committee at The University of Western Australia. All participants provided written informed consent. All participants were tested on their dominant hand, had normal or corrected-to-normal vision, and scored within the normal range (>24) on the Montreal Cognitive Assessment (Nasreddine, 2005). All PD patients were tested on-peak of their medication schedule.

The limited training condition was completed by seven PD patients (aged 59–78 years, 4 female) and nine older adult controls (aged 54–75 years, 5 female). All of these PD patients were on Levodopa (mean daily Levodopa dose: 408 ± 102 mg). Four of these PD patients were also on the dopamine agonist Pramipexole (mean daily dose 2.55 ± 0.67 mg). Disease duration ranged from 7 months to 8 years. PD patient severity rated according to the motor subscale of the Movement Disorders Society Sponsored Revised Unified PD Rating Scale (MDS-UPDRS) (Goetz et al., 2007) ranged from 7 to 30.

The extended training condition was completed by nine PD patients (aged 52–79 years, 3 female) and nine older adult controls (aged 59–77 years, 6 female). Eight of these PD patients were on Levodopa (mean daily Levodopa dose 472 ± 257), and four of these PD patients were also on the dopamine agonist Pramipexole (mean daily dose: 2.2 ± 0.9 mg). Disease duration ranged from 6 months to 9 years, and MDS-UPDRS motor subscale scores ranged from 10 to 44.

### Apparatus

Participants were seated on a height-adjustable chair in front of a laptop computer placed ~50 cm away from the participant along their midline. Participants held a digitizing pen (15.95 cm long, 1.4 cm wide, 17 g) on a WACOM Intuos 2 digitizing tablet (size: 30.48 cm × 30.48 cm, resolution ±0.025 mm). The pen's position on the tablet (XY coordinates) was sampled at 100 Hz and displayed on the computer monitor as a circular cursor with a 5 pixel radius (1.25 mm). Direct vision of the hand was prevented by placing the tablet and the hand directly beneath a stand, with the laptop placed atop the stand.

### General experimental procedure

The experimental task required participants to move the on-screen cursor from a start circle to a target circle by moving the digitizing pen on the digitizing tablet. Participants were first instructed to move a cursor representing the pen's position into the start circle. After the cursor came within the start circle for 2 s, a single-target circle of radius 23 pixels (6.08 mm) appeared 75 mm at 45° from the target. This single-target was used throughout the task. A tone sounded immediately after the target circle appeared, signaling participants to move the cursor to the target. Participants were instructed to move the cursor from the start circle to the target circle as accurately and as quickly as possible, in a single, uncorrected movement. Visual feedback of the movement trajectory was shown on-screen in real-time, and remained on-screen for 1 s after movement completion.

### Experimental design

Prior to adaptation, all participants completed a minimum of 30 baseline trials with veridical feedback, until three out of four consecutive movements were made with directional error of less than or equal to 3° and movement time was less than 1000 ms. Once this criteria were met, the test phase commenced. At the beginning of the test phase, participants completed a first block (A1) of either 25 (limited training condition) or 80 adaptation trials (extended training condition) in which visual feedback was rotated 30° counter-clockwise relative to the start circle. To compensate for the rotation, participants had to move in the 30° clockwise direction relative to the original movement direction. Previous work shows that 66 trials (per target) in A1 was sufficient to result in A1-B-A2 savings (Krakauer et al., 2005), and thus 80 trials with a single-target in A1was thought to constitute sufficient overlearning to evoke A1-B-A2 savings in controls. Participants then completed a second block of 25 adaptation trials with an opposing 30° clockwise rotation of visual feedback (B), such that to completely compensate for the rotation, participants had to move in the 30° counter-clockwise direction. Participants subsequently deadapted with 15 washout trials with veridical feedback. Previous work indicates that 15 washout trials were sufficient for directional error to reduce to pre-perturbation levels (Leow et al., 2012). In the ensuing third adaptation block A2, participants completed another 25 adaptation trials with the 30° counter-clockwise rotation previously experienced in A1. Finally, participants completed a further 15 washout trials with veridical feedback.

### Data analysis

Cartesian XY coordinates were recorded and used to plot movement trajectory. Directional error was scored at either (1) 100 ms into the movement after moving at least 5 mm (Bedard and Sanes, 2011) or (2) at 25% of movement trajectory, whichever came earlier. Directional error was calculated as the angular difference between this movement direction and an idealized movement direction starting from the start circle to the target circle. A negative value in directional error indicates that the on-screen movement trajectory was counter-clockwise to an ideal movement trajectory plotted from the start to the target, while a positive value denotes the opposite. To examine anterograde interference, it was necessary to compare negatively signed directional error in A to positively signed directional error in B. Thus, positively signed directional errors in B were converted to corresponding negatively signed values. A single-exponential function was fit to the group mean trial-by-trial directional error for each adaptation block for graphical depiction.

Savings and anterograde interference were quantified by examining block-to-block changes in percent adaptation calculated from the rapid error reduction phase (taken as Trials 2–15) of each block (Leow et al., 2012). The first trial of each block was not considered as there is no opportunity to correct error on the first trial. The method of evaluating block-to-block changes using percent adaptation in the rapid error reduction phase has been previously validated (Krakauer et al., 2005). Percent adaptation was computed with the formula: Percent adaptation = 100% × [1 − (Mean directional error/30°)]. Mean directional error was calculated from the mean of directional error in Trials 2–15, as rapid error reduction occurred in Trials 2–15 in the current study. Mixed ANOVAs and paired *t*-tests were used to evaluate block-to-block changes in percent adaptation within each participant group. Where applicable, Bonferroni corrections were used to correct for violations of sphericity. Effect sizes were quantified using η^2^ and Cohen's *d*. By convention, η^2^ values were categorized as: 0.01~ small, 0.06~ medium, 0.14~ large, and Cohen's *d*-values were categorized as: 0.20~ small, 0.50~ medium, 0.80~ large. Block-to-block changes in percent adaptation were reported as means ± standard errors of the mean. A1-B-A2 savings was quantified by increased percent adaptation from A1 to A2. Anterograde interference was quantified by decreased percent adaptation from A1 to B.

It is noted that other studies quantify anterograde interference by comparing performance in B in a group that has completed A1 to performance in B of a control group that did not previously complete A1 (Cothros et al., 2006). However, the current method of quantifying anterograde interference by comparing adaptation performance in B with that in A1 has been shown to be a sensitive measure of anterograde interference (Sing and Smith, 2010).

## Results

### PD patients show similar rate and extent of A1 error reduction

**Figure 1:**
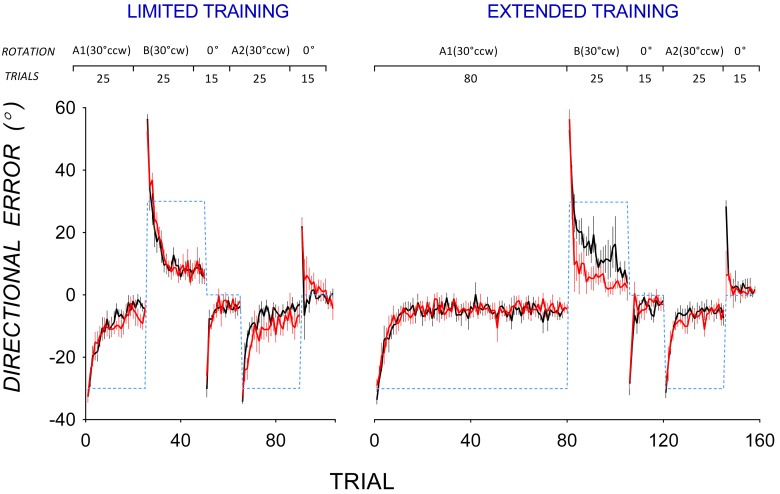
**Mean trial-by-trial directional error during the adaptation phase for the limited training (top) and extended training (bottom) conditions in PD patients (black lines) and controls (red lines).** Error bars show standard errors of the mean. Dotted lines show the rotation of visual feedback in each phase. The rotation of feedback (CCW, counter-clockwise; CW, clockwise) and the number of trials in each phase is shown at the top.

Figure 1 shows group mean trial-by-trial directional error in all adaptation phases in PD patients (red lines) and controls (black lines) for the limited (left panel) and the extended training condition (right panel). In A1, PD patients and controls appeared to reduce directional error at a similar rate in both the limited and extended training conditions. Mixed ANOVAs with between-subjects factor Group (PD, controls) and within-subjects factor Trial (Trials 1–25) were run separately for the limited and the extended training condition. In both analyses, there was no significant main effect of Group, and no significant Group by Trial interaction. To evaluate if PD patients and controls differed in the extent of error reduction in A1, mixed ANOVAs with between-subjects factor Group (PD, controls) and within-subjects factor (Trials 16–25) were run separately for the limited and extended training conditions. These trials were selected to estimate asymptotic directional error as little further error reduction occurred beyond Trial 16. In the limited training condition, there was no significant main effect of Group [*F*_(1, 14)_ = 1.33, *p* = 0.3, η^2^ = 0.09], and no significant Group by Trial interaction [*F*_(5.0, 69.8)_ = 0.93, *p* = 0.5, η^2^ = 0.07]. Similarly, in the extended training condition, there was no significant main effect of Group [*F*_(1, 16)_ = 0.24, *p* = 0.6, η^2^ = 0.02], and no significant Group by Trial interaction [*F*_(4.8, 76.5)_ = 1.00, *p* = 0.4, η^2^ = 0.06]. Hence PD patients and controls did not differ in the extent of adaptation in A1 in either the limited or the extended training condition. To evaluate if PD patients differed from controls in variability of directional error at asymptote in A1, trial-by-trial variability of directional error at asymptote in A1 was estimated using standard deviations calculated from Trials 16–25 of A1. Variability of directional error at asymptote did not differ significantly between PD patients and controls in either the limited [*F*_(1, 14)_ = 2.06, *p* = 0.2, η^2^ = 0.13] or the extended training condition [*F*_(1, 17)_ = 0.66, *p* = 0.4, η^2^ = 0.04].

### Impaired A1-B-A2 savings in PD

**Figure 2:**
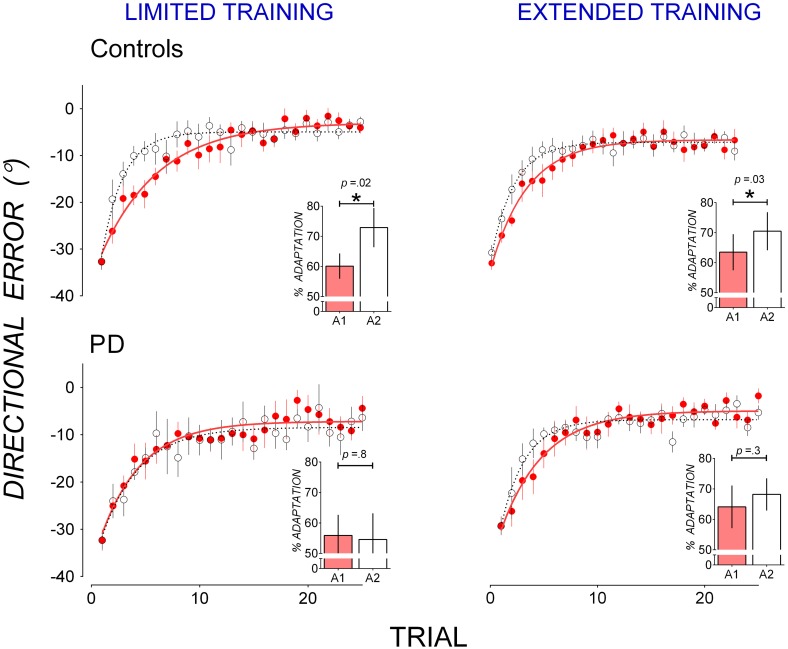
**Mean trial-by-trial directional error in A1 (closed, red circles) and A2 (open circles) in controls (top panels) and PD patients (bottom panels) for the limited training condition (left panels) and the extended training condition (right panels).** A single-exponential function was fit to group mean trial-by-trial directional error for each adaptation block in A1 (solid lines) and A2 (broken lines). Insets: percent adaptation calculated from mean directional error of Trials 2–15 for each adaptation block. Error bars represent standard errors of the mean.

Figure 1 also shows that in A2, mean directional error in PD patients appeared to decrease more slowly than in controls after both limited and extended training in A1. To facilitate comparison of savings, data from A1 and A2 are replotted in Figure 2. Controls reduced directional error more rapidly in A2 (open circles) than in A1 (closed circles) in both the limited (Figure 2 top left panel) and the extended training condition (Figure 2 top right panel), indicating A1-B-A2 savings. PD patients showed similar rates of error reduction in A1 and A2 in both the limited (Figure 2 bottom left panel) and the extended training condition (Figure 2 bottom right panel) indicating a lack of A1-B-A2 savings.

Percent adaptation averaged from Trials 2–15 of A1 (filled bars) and A2 (clear bars) are shown in Figure 2 insets. To evaluate the effect of participant group and training on block-to-block changes in percent adaptation, a mixed-ANOVA with between-subjects factors Group (PD, controls) and Training (Limited, Extended) and within-subjects factors Block (Block A1 and A2) was run on percent adaptation data. The Group by Block interaction [*F*_(1, 30)_ = 3.78, *p* = 0.06, η^2^ = 0.09] suggests that controls and PD patients might have differed in the way percent adaptation changed from Block A1 to A2. *T*-tests showed that in the limited training condition, controls significantly increased percent adaptation from A1 to A2 [*t*_(8)_ = 2.78, *p* = 0.02, *d* = 0.71, mean increase: 12.73 ± 4.58%], but PD patients did not [*t*_(6)_ = 0.19, *p* = 0.8, *d* = 0.08, mean increase: 1.26 ± 6.60%]. Similarly in the extended training condition, controls significantly increased percent adaptation from A1 to A2 [*t*_(8)_ = 2.43, *p* = 0.034, *d* = 0.40, mean increase: 6.98 ± 2.87%], but PD patients did not [*t*_(8)_ = 1.11, *p* = 0.3, *d* = 0.21, mean increase: 3.94 ± 3.56%]. Hence while controls showed significant A1-B-A2 savings after both limited and extended training, PD patients did not show significant A1-B-A2 savings after either limited or extended training.

### Impaired anterograde interference in PD

**Figure 3:**
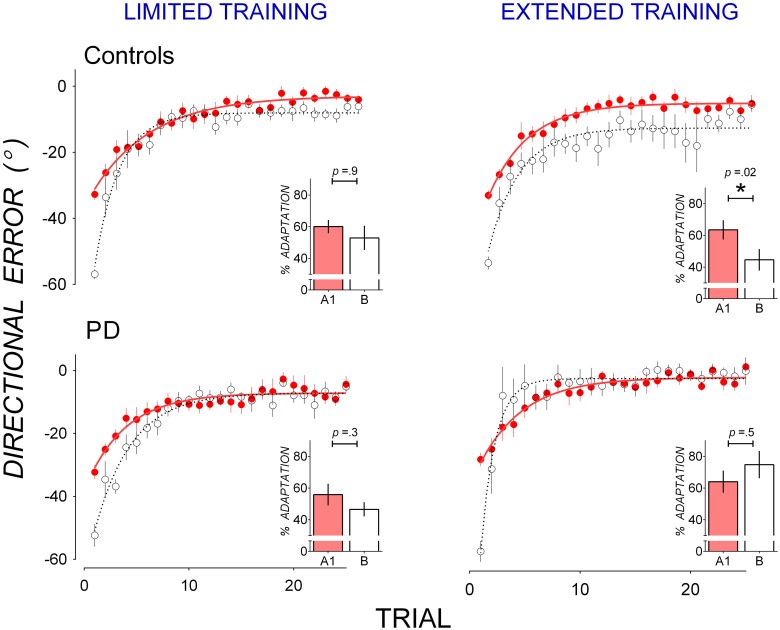
**Mean trial-by-trial directional error in A1 (closed, red circles) and B (open circles) in controls (top panels) and PD (bottom panels) for the limited training condition (left panels) and the extended training condition (right panels).** A single-exponential function was fit to group mean trial-by-trial directional error for A1 (solid lines) and B (broken lines). Insets: percent adaptation calculated from mean directional error of Trials 2–15 for each adaptation block. Error bars represent standard errors of the mean.

Figure 1 shows that PD patients reduced directional error more quickly than controls in B after extended training in A1, suggesting that PD patients showed less anterograde interference from A1 to B than controls. Mean trial-by-trial directional error of A1 and B are replotted in Figure 3 to facilitate comparison of anterograde interference. Both PD patients (bottom panels) and controls (top panels) showed large directional error in the first trial of B of approximately twice the magnitude of directional error in the first trial of A1, thus reflecting the change in rotation from 30° counter-clockwise in A1 to 30° clockwise in B. After limited training in A1 (Figure 3, left panels), both controls (top panel), and PD patients (bottom panel) showed similar rates of error reduction in A1 and B, indicating little anterograde interference from A1 to B. After extended training in A1, however, controls showed greater error in B than in A1 (Figure 3, top right panel), indicating anterograde interference, whereas PD patients did not (Figure 3, bottom right panel), indicating little anterograde interference.

Anterograde interference was quantified as a reduction in percent adaptation averaged from Trials 2–15 of Block A1 and B, nd is shown in insets in Figure 3. These scores were subjected to mixed ANOVAs with between-subjects factors Group (Controls, PD) and Training (Limited, Extended) and within-subjects factors Block (A1, B). A significant Group by Block by Training interaction [*F*_(1, 30)_ = 4.67, *p* = 0.04, η^2^ = 0.11] suggests that groups differed in block to block changes in percent adaptation depending on training condition. This Group by Block by Training interaction was followed up with mixed-ANOVAs with a between-subjects factor Group (PD, Controls) and a within-subjects factor Block (A1, B) run separately for the limited and the extended training conditions.

In the limited training condition, neither the main effect of Block [*F*_(1, 14)_ = 3.12, *p* = 0.09, η^2^ = 0.15] nor the main effect of Group [*F*_(1, 14)_ = 0.53, *p* = 0.5, η^2^ = 0.04] or their interaction [*F*_(1, 14)_ = 0.05, *p* = 0.8, η^2^ = 0.00] were significant. Both controls (Figure 3 top left panel inset) and PD patients (Figure 3 bottom left panel inset) showed little reduction in percent adaptation from A1 to B, suggesting a lack of anterograde interference. Percent adaptation did not decrease significantly from A1 to B in either the control group [*t*_(8)_ = 0.09, *p* = 0.9, *d* = 0.02, mean reduction: 7.21 ± 5.89%], or the PD group [*t*_(6)_ = 1.25, *p* = 0.3, *d* = 0.61, mean reduction: 9.25 ± 7.38%].

In the extended training condition, there was a significant Group by Block interaction [*F*_(1, 16)_ = 6.74, *p* = 0.01, η^2^ = 0.24] which resulted from a decrease in percent adaptation from A1 to B in the control group (Figure 3, top right panel inset) but not in the PD group (Figure 3, bottom right panel inset). The decrease in percent adaptation from A1 to B was significant in the controls [*t*_(8)_ = 2.93, *p* = 0.02, *d* = 1.36, mean decrease: 26.43 ± 9.13%], showing anterograde interference. The decrease in percent adaptation was not significant in the PD group [*t*_(8)_ = 0.67, *p* = 0.5, *d* = 0.23, mean decrease: 7.35 ± 8.80%], showing a lack of anterograde interference. Hence extended training in A1 evoked significant anterograde interference in controls but not in PD patients. The top right panel in Figure 3 shows that, for controls after extended in A1, directional error was greater in B than A1 not only in Trials 2–15, but also in Trials 16–25 where little further error reduction occurred. This suggests anterograde interference was not limited to the rapid error reduction phase, but persisted through the asymptotic phase. To evaluate this possibility, asymptotic directional error was estimated by averaging Trials 16–25 of adaptation block A1 and B for each dataset. Mean asymptotic error was larger in B (−11.65 ± 10.08°) than in A1 (−5.33 ± −4.28°) in these trials and this difference approached significance [*t*_(8)_ = 1.55, *p* = 0.07, one-tailed], with a moderate effect size (*d* = 0.72).

## Discussion

The current study yielded two main findings. First, controls showed savings after both limited and extended training in A1, but showed anterograde interference after extended but not limited training in A1. Second, PD patients did not show anterograde interference or savings after either limited or extended training in A1. These results indicate that different mechanisms contribute to savings and anterograde interference, and that these mechanisms are both impaired in PD.

### Different mechanisms contribute to anterograde interference and savings

The current data show that savings and anterograde interference require different amounts of training. A limited training regime of 25 trials was sufficient to elicit savings, but not anterograde interference. That extended training was necessary to elicit anterograde interference but not savings shows that a two-state model comprising a fast and a slow process (Smith et al., 2006) cannot account for both savings and anterograde interference. If the same mechanism(s) in this model contributes to both savings and anterograde interference, the same amount of training should produce both savings and anterograde interference. We suggest that the model-free mechanisms of reinforcement learning and use-dependent plasticity have different training requirements and show different contributions to anterograde interference and savings: while limited training might be sufficient to engage reinforcement mechanisms responsible for savings, extended training might be necessary to additionally engage other mechanisms to elicit anterograde interference. Use-dependent plasticity is a plausible candidate, as it requires extended movement repetition (Classen et al., 1998). Extended training with a single-target in A1 entailed extended repetition of a single adapted movement, likely generating a use-dependent bias in the same direction, thus slowing error reduction in B.

Savings has been attributed to reinforcement mechanisms which associate the adapted movement with reward at initial learning such that the adapted movement is preferentially selected when relearning the same perturbation, speeding up adaptation (Huang et al., 2011). Limited training of 25 trials thus appears sufficient to engage this reinforcement mechanism. At first glance, this finding seems inconsistent with Krakauer et al. (2005) who found that 33 cycles in A1 (33 visits to each of 8 different targets) was insufficient to elicit A1-B-A2 savings. This could be due to the different number of targets used: in the current single-target design, a single adapted movement was reinforced, whereas in the multiple-target design of Krakauer et al. (2005), multiple movements to spatially separated targets were reinforced. Reinforcement mechanisms may be more effective in a single-target design where the adapted movement is repeated and reinforced in consecutive trials than in a multiple-target design.

Our results also indicate that savings is unlikely to result from use-dependent plasticity mechanisms, because repetition-induced movement biases should have been eliminated by the washout trials prior to A2. This is consistent with previous findings showing that use-dependent plasticity alone is insufficient for savings. For instance, repeating a movement in the direction of an ideally adapted movement in the absence of a perturbation failed to elicit savings in subsequent adaptation (Huang et al., 2011). Furthermore, use-dependent plasticity might not be crucial to savings, as savings is not decreased when repetition of the fully adapted movement is reduced via a gradual adaptation schedule (Klassen et al., 2005), or even when repetition of the adapted movement is prevented completely (Huang et al., 2011).

It is not thought that use-dependent plasticity alone is sufficient to elicit anterograde interference. Findings of anterograde interference even with a 24-h delay between A1 and B (Cothros et al., 2006) appear inconsistent with the suggestion that use-dependent plasticity alone is responsible for anterograde interference, as use-dependent movement biases typically decay after 60 min (Classen et al., 1998). Reinforcement mechanisms likely to contributes to anterograde interference: a rewarding outcome resulting from execution of the adapted movement reinforces that movement such that it is preferentially selected even when the perturbation in subsequent learning opposes that in initial learning, slowing the rate of subsequent learning (Huang et al., 2011).

It is noteworthy that anterograde interference in controls was not only evident in the error reduction phase, but also in the asymptotic phase, where directional error remained larger in B than in A1. This phenomenon has previously been observed (Tong and Flanagan, 2003; Cothros et al., 2006; Sing and Smith, 2010; Zach et al., 2012), but has received little attention. Larger asymptotic error in B cannot be completely attributed to use-dependent plasticity as it was also evident when repetition of movement to a single direction was prevented by a multiple-target design (Tong and Flanagan, 2003; Cothros et al., 2006; Zach et al., 2012). The persistence of the previously reinforced movement in A1 could additionally contribute to larger asymptotic error in B. This proposal is consistent with a recent finding that reinforcing an adapted movement without error feedback during asymptote increases persistence of that movement in subsequent error clamp trials (Shmuelof et al., 2012). We therefore suggest that both use-dependent and reinforcement mechanisms elicited from extended training contribute to anterograde interference.

### Savings and anterograde interference are both impaired in PD

Unlike controls, who showed savings after both limited and extended training, PD patients did not show A1-B-A2 savings after either limited or extended training. This is the first time that impaired A1-B-A2 savings in PD has been demonstrated, and this extends previous findings of impaired savings in PD with an A1-washout-A2 paradigm (Marinelli et al., 2009; Bedard and Sanes, 2011; Leow et al., 2012). Dopamine dysfunction and consequently deficient reinforcement mechanisms in PD may result in difficulty associating the adapted movement for A as well as the adapted movement for B with reward, such that in A2, the adapted movement for A is not preferentially selected, attenuating savings. On the other hand, the finding of impaired anterograde interference in PD is novel, and suggests that intact dopaminergic function is important to the use-dependent plasticity mechanisms thought to contribute to anterograde interference.

Dopaminergic treatment in PD patients often overdoses the relatively unaffected ventral striatum while treating the more affected dorsal striatum (for a review, see Cools, 2006). While impaired savings has been shown even in drug-naïve PD patients who are unaffected by dopamine medication overdose effects (Marinelli et al., 2009), the current findings of impaired anterograde interference in medicated PD patients could result from overdosing the less affected ventral striatum. Future studies examining anterograde interference in drug-naïve PD patients should clarify if dopamine denervation alone can impair anterograde interference.

It is important to bear in mind that reinforcement and use-dependent mechanisms were not directly manipulated in this study. Instead, the dopamine dysfunction in PD that impairs reinforcement and use-dependent plasticity mechanisms was used to explore the role of these mechanisms in savings and interference. Our interpretation was built upon current knowledge of the role of reinforcement in adaptation learning (Diedrichsen et al., 2010; Huang et al., 2011; Izawa and Shadmehr, 2011; Pekny et al., 2011; Shmuelof et al., 2012), the role of dopamine in reinforcement (Frank, 2005) and use-dependent plasticity (Floel, 2005; Floel et al., 2008; Rösser et al., 2008). However, we cannot rule out the possibility that other mechanisms might additionally contribute to savings and interference.

### Potential neural mechanisms of savings and anterograde interference

The primary motor cortex (M1) has been shown to play an important role in savings and anterograde interference. While altering M1 activity during adaptation does not affect initial rate of adaptation learning, decreasing M1 excitability using repetitive transcranial magnetic stimulation selectively impaired both anterograde interference (Cothros et al., 2006) and savings (Riek et al., 2012), while increasing M1 excitability using transcranial direct current stimulation of M1 markedly increased retention of the learned visuomotor rotation (Galea et al., 2011). M1 is thought to encode a longer-term representation of motor adaptation, as repeating the adapted movement after attaining asymptote changes the preferred direction of a subgroup of M1 neurons to the adapted movement direction (Gandolfo et al., 2000; Li et al., 2001; Paz et al., 2003), and this change persists across test sessions spanning several days (Paz et al., 2003; Richardson et al., 2012), despite washout (Li et al., 2001; Paz et al., 2003) and subsequent adaptation to an opposing perturbation (Zach et al., 2012). However, it is unclear whether use-dependent and/or reinforcement mechanisms contribute to this phenomenon since there is at present no direct evidence supporting this suggestion. Future studies could elucidate if and how use-dependent plasticity and reinforcement mechanisms influence the longer-term representation of motor adaptation in M1 by systematically varying movement repetition and reward during adaptation while recording or disrupting M1 activity.

Midbrain dopaminergic signals to M1 may be important to both model-free slow mechanisms of reinforcement and use-dependent plasticity and might thus contribute to the formation of a longer-term representation of adaptation learning in M1. M1 is connected to the midbrain through indirect and direct projections (for a review, see Luft and Schwarz, 2009). Dopamine reward signals influence M1 activity, as M1 excitability is modulated by the probability of reward in neurologically intact adults but not in unmedicated PD patients (Kapogiannis et al., 2008, 2011). Midbrain dopaminergic signals influence the LTP-like processes thought responsible for use-dependent plasticity in M1 (Floel et al., 2008), and dopamine denervation in PD impairs M1 LTP-like plasticity in PD (Morgante et al., 2006; Suppa et al., 2011; Kishore et al., 2012). Hence blunted midbrain dopaminergic signals in PD resulting in attenuated modulation of M1 activity, might impair both reinforcement and use-dependent mechanisms.

## Summary

This study shows that in neurologically intact controls, extended training of 80 trials in A1 was necessary to elicit anterograde interference but not necessary to elicit A1-B-A2 savings, which was evident even after limited training of 25 trials in A1. We suggest that while reinforcement mechanisms evoked by limited training are sufficient to elicit A1-B-A2 savings, additional use-dependent plasticity mechanisms evoked by extended training is necessary to elicit anterograde interference. Furthermore, this study also shows that dopamine dysfunction in PD impairs both anterograde interference and A1-B-A2 savings, which suggests that dopamine is important to both reinforcement and use-dependent mechanisms activated during motor adaptation.

### Conflict of interest statement

The authors declare that the research was conducted in the absence of any commercial or financial relationships that could be construed as a potential conflict of interest.
